# Chronic uterine inversion presenting with severe anemia 7 years after a home delivery and the subsequent successful pregnancy: a case report

**DOI:** 10.1186/s13256-023-04012-x

**Published:** 2023-07-04

**Authors:** Fekata Defere Tolcha, Tufa Bube Dale, Tilahun Tufa Anbessie

**Affiliations:** 1 Madda Walabu University, Goba Referral Hospital, School of Medicine, Goba, Oromia Ethiopia; 2 Department of Obstetrics and Gynecology, Madda Walabu University, Goba Referral Hospital, Goba, Oromia Ethiopia; 3 Department of Public Health, Madda Walabu University, Goba Referral Hospital, Goba, Oromia Ethiopia

**Keywords:** Chronic uterine inversion, Severe anemia, Successful delivery, Subsequent pregnancy, Case report

## Abstract

**Background:**

Uterine inversion is a clinical condition characterized by the folding of the uterine fundus into the uterine cavity or beyond the cervix. Chronic uterine inversions that manifest seven years after delivery are extremely rare, despite the fact that both acute and chronic uterine inversions are infrequent. Unlike uterine inversion during parturition, which can be promptly managed, chronic uterine inversion poses a diagnostic and management challenge. We, herein, report a patient who was managed and followed at our institution for chronic uterine inversion.

**Case presentation:**

A 28-year-old African female who was referred to our institution with complaints of secondary infertility for seven years, abnormal vaginal bleeding, and lower abdominal pain for 12 months with a mass-like sensation in the vagina. At presentation, she had pale conjunctiva and a protruded, rubbery mass in the cervix with indistinguishable cervical OS on vaginal examination. The patient was resuscitated with intravenous fluids and three units of blood, after which Haultain’s procedure was performed. After 16 months on a contraceptive, she was able to conceive and deliver a healthy neonate.

**Conclusion:**

Severe anemia can rarely be a presenting symptom of chronic uterine inversion. Following a surgical procedure for chronic uterus inversion, a successful delivery is possible if thorough follow-up is carried out.

## Background

Uterine inversion is an abnormal protrusion of the uterus through the uterine cavity. Acute and chronic uterine inversions are life-threatening, but the former one, occurring as a postpartum complication, can lead to massive hemorrhage, which can be fatal in the third stage of labor [1]. Chronic uterine inversion is a rare disorder that is usually observed after 4 weeks of delivery. In addition to causes like leiomyoma and carcinoma, in developing countries like India and Ethiopia, where home deliveries are still practiced, chronic uterine inversion is encountered as a missed incomplete uterine inversion. In contrast to acute uterine inversion, symptoms are usually subtle and become clinically apparent with time [2].

Surgical interventions involving the uterine wall can predispose patients to several complications, including uterine rupture and uterine dehiscence in subsequent pregnancies. Operative procedures to correct uterine inversion incur an additional risk of uterine re-inversion. There are reports of uterine preservation for subsequent fertility after the management of chronic puerperal uterine inversion, but very few successful deliveries have been reported [3]. The peculiarity of our case stems from two factors. First, chronic uterine inversion presenting with severe anemia seven years postpartum is extremely rare. In addition, the patient was able to conceive and deliver successfully through spontaneous vaginal delivery without a complication. Hence, we hope this case report will shed some light on future conception plans for chronic uterine inversion and strengthen the existing scientific validity of the management procedure.

## Case presentation

This is a 28-year-old African para1 housewife who presented with symptoms of irregular menses, vaginal bleeding, and lower abdominal pain of one-year duration. Seven years prior, she gave birth at home with the assistance of a traditional birth attendant who had difficulty delivering the placenta. After the delivery, both the mother and neonate were in good health and did not visit a hospital. Our patient had no previous history of gynecologic surgery, sexual disorders, pelvic inflammatory disease, or abortions. She received an HPV vaccination. There were no intermenstrual or postictal bleedings, and her menses, which last 3–5 days, occur every 28–30 days.

She has had normal menses for the six years following the delivery, but she has not been able to conceive. She was examined for infertility at a private clinic, where she was informed of normal results from hormonal (LH, FSH) and abdominopelvic ultrasound studies. She experienced a mass-like sensation in the vagina, abdominal pain, and vaginal bleeding 2 months before presenting to our hospital. She was diagnosed with AUB (abnormal uterine bleeding) after being investigated with a CBC (complete blood count), which showed moderate anemia, at a local health center. Despite being given iron tablets and a combined oral contraceptive, her symptoms had not improved. In addition, she had no family history of bleeding diathesis, diabetes, heart disease, or any previous gynecological procedures.

Upon admission, the patient appeared pale and acutely sick, with vital signs showing a pulse rate of 112 beats per min and a blood pressure (BP) of 90/60 mmHg. On physical examination, she had pale conjunctiva and a soft and non-tender abdomen. PV examination showed there was a rubbery mass protruding through the cervical OS, which made it difficult to pass the examining finger through the cervical rim.

**Fig. 1:**
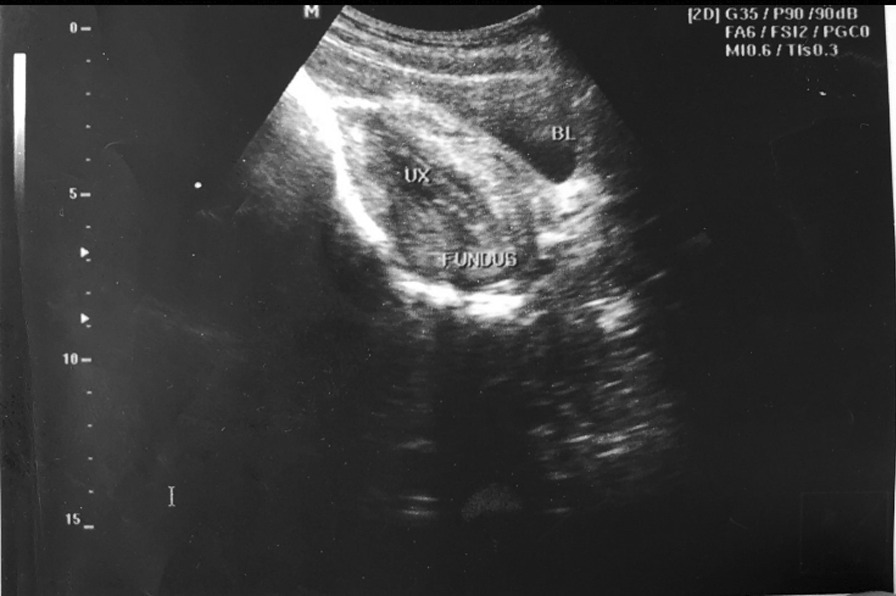
Pelvic ultrasound depicting uterine fundus at cervical OS. UX, Uterus; BL, Bladder; Fundus, Uterine Fundus

On investigation, her hemoglobin and hematocrit were 3.9 g/dl and 12%, respectively. Pelvic ultrasound revealed the uterine fundus to be on cervical OS, as depicted in Fig. 1, pointing towards the diagnosis of uterine inversion. There were no tumors or fibroids on the pelvic ultrasound.

**Fig. 2:**
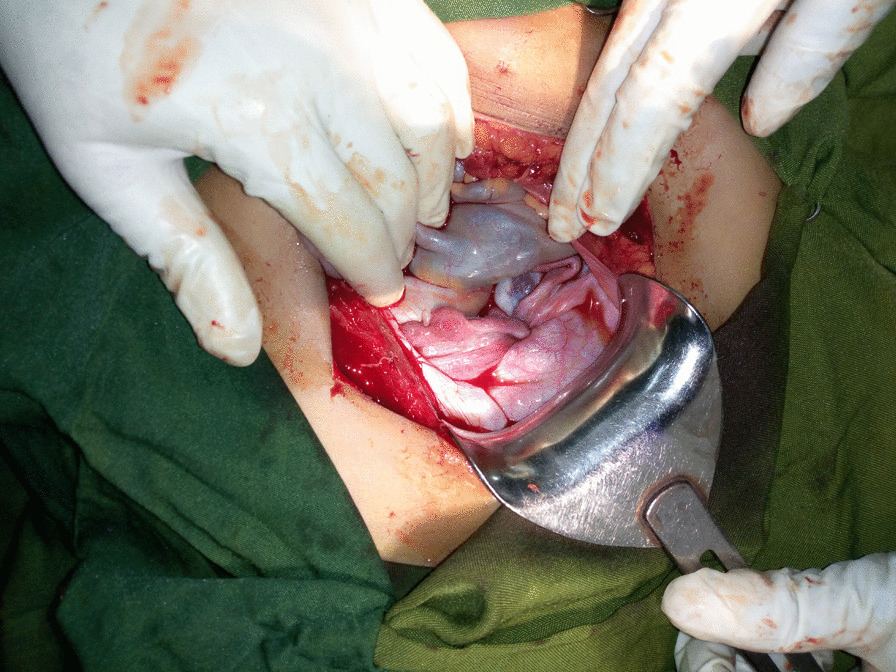
Intraoperative picture showing cupping of the uterus and inward pulled ovaries and tubes

**Fig. 3:**
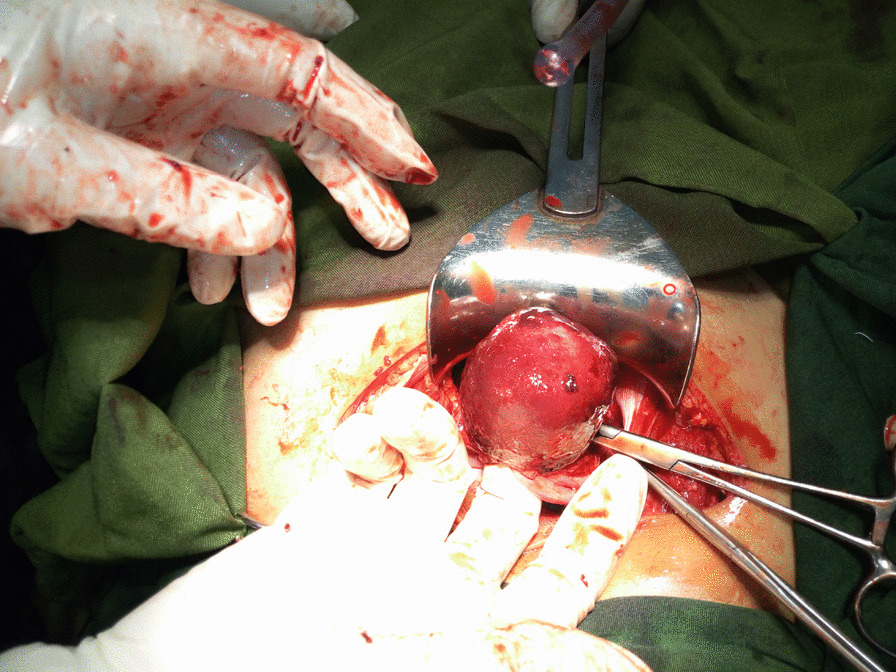
Intraoperative picture showing repositioning of the uterus with the surgeon’s finger lifting the fundus upward

After transfusing 3 units of whole blood, the patient was prepared for surgery, and the first step was to try to manually return the fundus into the uterine cavity, which yielded no success. Under general anesthesia, the abdomen was opened by a Pfannenstiel incision, where a typical flower vase appearance with fundal cupping of the uterus and inward pulling of the tubes and ovaries was seen as in Fig. 2. Hence, confirming the diagnosis of uterine inversion. The uterus was held at the level of the bilateral round ligaments, and a vertical incision was made to the posterior wall at the point of the constrictor ring. Finally, the uterus was gently lifted upward and repositioned as depicted in Fig. 3, and the normal anatomy of the pelvic organs was restored by Haultain’s technique. Apart from the inversion, there were no masses or abnormal findings on the uterus.

Our patient’s post-operative period was uneventful, with no complications and stable observations. Investigations (a renal function test and a liver function test) done after the surgery were within the normal range. Upon correction of the post-operative hemoglobin and hematocrit to 10 g/dl and 29%, respectively, she was discharged with iron tablets and advised on the next delivery. In addition to being given a subdermal implant, we stressed the need to deliver the next child by elective cesarean delivery. At her follow-up visit at 4 weeks, the patient was well, with normal vital signs and no bleeding. On a subsequent visit in the third month, the patient reported no new complaints, and menstrual flow was normalized.

The patient returned 16 months after the procedure and informed us of her desire to conceive. Upon removal of the contraceptive, we advised her to adhere to strict follow-up. She conceived 4 months later, and her follow-up antenatal care was uneventful. At 37 weeks, the patient started to have labor pains and was unable to reach a hospital in time. By the time the ambulance arrived at her home, she was in the second stage of labor and had delivered via spontaneous vaginal delivery on the way to a health facility. The placenta was removed carefully by a midwife once she arrived at the hospital, and there was no excessive bleeding or derangement of the vital signs. Abdominal ultrasound, performed in the immediate postpartum period and on subsequent follow-up after 3 months, showed the uterus in its normal position.

## Discussion

Uterine inversion is a rare clinical entity that is typified by the invagination of the uterine fundus and/or corpus through the uterine cavity. It can be classified as puerperal and non-puerperal based on the cause of inversion or complete and incomplete depending on the position of the uterine fundus. Furthermore, puerperal inversion can be grouped based on the time difference between parturition and the presentation of inversion. We consider inversion acute if it occurs within the first 24 h of delivery, sub-acute (between 24 h and 4 weeks), and chronic (at least after 4 weeks) with prevalence of 83.4%, 2.62%, and 13.9%, respectively [4, 5]. Our case was a chronic uterine inversion presenting seven years after a home delivery.

The commonest cause of acute uterine inversion is mismanagement of the third stage of labor, while leiomyoma is the major culprit for chronic non-puerperal uterine inversion, accounting for 56.2% [3]. One of the causes of chronic uterine inversion is incomplete puerperal inversion, which could be missed at delivery and present any time after 4 weeks. Home deliveries are common in developing countries, which may lead to uterine inversion as mismanagement may occur due to the limited knowledge of the untrained birth attendant [6]. The exact mechanism by which inversion occurs is still not well studied. However, factors like excessive traction during placental delivery in an atonic uterus, inappropriate fundal pressure, multiparty, dragging effects from an intrauterine mass, and a sturdy umbilical cord can increase the risk of inversion [7].

As for our patient, her first delivery was a home delivery complicated by an untrained birth attendant seven years’ ago. Since then, she has visited hospitals with abnormal vaginal bleeding and an inability to conceive before finally being referred to our hospital. Hence, mismanaged labor caused incomplete uterine inversion, which later presented as chronic uterine inversion with severe anemia.

Uterine inversion may present with a wide range of signs and symptoms. If acute, it manifests with life-threatening post-partum bleeding, hypovolemic shock, and if not promptly managed, it can result in maternal death. If it is chronic, it may present with a sensation of mass in the cervix or vagina, abnormal vaginal bleeding, vaginal discharge, chronic pelvic pain, and infertility. Furthermore, there may be a mass visible in the vaginal canal. Since chronic uterine inversion poses a challenge in its diagnosis and management, investigations like ultrasonography and MRI are helpful [8]. Abnormal vaginal bleeding, secondary infertility, and severe anemia (a very rare presentation in chronic uterine inversion) were our patients’ presentations. A physical examination and abdominal pelvic ultrasound suggested uterine inversion, which was confirmed intraoperatively.

There are a few surgical methods for reducing both acute and chronic uterine inversion. Among those procedures, the Haultain procedure was the one with the highest success rate [3]. The Huntington and Haultain techniques are commonly used for abdominal procedures. Huntington’s procedure involves grasping the round ligaments and the uterus below the area of inversion and slowly pulling up repeatedly until the uterus is re-inverted [9]. Haultain’s procedure involves incising the posterior of the vaginal-cervical ring and carrying up the posterior wall of the uterus until it is re-inverted to its normal anatomy [10]. Kustner and Spinelli’s vaginal approach procedures could also be used. We were able to successfully correct uterine anatomy using Haultain’s technique.

The increased risk of uterine dehiscence, rupture, or re-inversion after uterine incision increases the likelihood of a cesarean section, especially if the procedure involves vertical uterine incisions. There are few reports of successful pregnancies following the surgical correction of puerperal uterine inversion [3]. Post-surgical deliveries are almost always by cesarean section, as it decreases the risk of uterine rupture and re-inversion. In our case, due to factors like lack of transportation and the fast progression of labor, she delivered through spontaneous vaginal delivery, which is not recommended. Although it was a precipitous labor, the third stage of labor and post-partum were carefully managed to prevent re-inversion.

## Conclusion

Chronic uterine inversion is a rare condition that can be challenging to diagnose and manage. It presents with abnormal uterine bleeding, secondary infertility, and severe anemia in patients who had difficult placental separation on previous deliveries. Despite its rarity, health care professionals should strongly consider chronic uterine inversion in a patient presenting with that set of symptoms. If timely and appropriately managed for uterine inversion, it is possible to successfully conceive after 12 months of post-operative period given the appropriate adherence to follow-up. If uterine inversion is reversed with the Haultain procedure, patients should be put on contraception for at least a year before trying to get pregnant. Delivery should be by cesarean section, and strict puerperal follow-up is imperative to prevent re-inversion.

## Data Availability

All data and materials for this case report are available from the corresponding author upon request by the journal.
